# Baseline Natural Killer and T Cell Populations Correlation with Virologic Outcome after Regimen Simplification to Atazanavir/Ritonavir Alone (ACTG 5201)

**DOI:** 10.1371/journal.pone.0095524

**Published:** 2014-05-06

**Authors:** John E. McKinnon, Robbie B. Mailliard, Susan Swindells, Timothy J. Wilkin, LuAnn Borowski, Jillian M. Roper, Barbara Bastow, Mary Kearney, Ann Wiegand, John W. Mellors, Charles R. Rinaldo, for the A5201 study team

**Affiliations:** 1 Henry Ford Hospital System, Detroit, Michigan, United States of America; 2 University of Pittsburgh, Pittsburgh, Pennsylvania, United States of America; 3 University of Nebraska Medical Center, Omaha, Nebraska, United States of America; 4 Weill-Cornell Medical College, New York, New York, United States of America; 5 George Washington University, Washington, DC, United States of America; 6 Social & Scientific Systems, Inc., Silver Spring, Maryland, United States of America; 7 HIV Drug Resistance Program, NCI, Frederick, Maryland, United States of America; University of Missouri-Kansas City, United States of America

## Abstract

**Objectives:**

Simplified maintenance therapy with ritonavir-boosted atazanavir (ATV/r) provides an alternative treatment option for HIV-1 infection that spares nucleoside analogs (NRTI) for future use and decreased toxicity. We hypothesized that the level of immune activation (IA) and recovery of lymphocyte populations could influence virologic outcomes after regimen simplification.

**Methods:**

Thirty-four participants with virologic suppression ≥48 weeks on antiretroviral therapy (2 NRTI plus protease inhibitor) were switched to ATV/r alone in the context of the ACTG 5201 clinical trial. Flow cytometric analyses were performed on PBMC isolated from 25 patients with available samples, of which 24 had lymphocyte recovery sufficient for this study. Assessments included enumeration of T-cells (CD4/CD8), natural killer (NK) (CD3^+^CD56^+^CD16^+^) cells and cell-associated markers (HLA-DR, CD's 38/69/94/95/158/279).

**Results:**

Eight of the 24 patients had at least one plasma HIV-1 RNA level (VL) >50 copies/mL during the study. NK cell levels below the group median of 7.1% at study entry were associated with development of VL >50 copies/mL following simplification by regression and survival analyses (p = 0.043 and 0.023), with an odds ratio of 10.3 (95% CI: 1.92–55.3). Simplification was associated with transient increases in naïve and CD25^+^ CD4^+^ T-cells, and had no impact on IA levels.

**Conclusions:**

Lower NK cell levels prior to regimen simplification were predictive of virologic rebound after discontinuation of nucleoside analogs. Regimen simplification did not have a sustained impact on markers of IA or T lymphocyte populations in 48 weeks of clinical monitoring.

**Trial Registration:**

ClinicalTrials.gov NCT00084019

## Introduction

Trials of antiretroviral treatment (ART) simplification to ritonavir-boosted protease inhibitors (PI) alone have shown mixed success, with some trials mirroring the outcome of standard triple therapy and others failing to show equivalence.[1], [2] The criteria for selection of patients for treatment simplification trials have varied and include baseline CD4 T-cell counts, duration of prior suppressive antiretroviral therapy and use of specific antiretroviral agents, making it difficult to compare studies in order to identify predictors of virologic outcome. Prior analyses have identified duration of suppressive ART, low hemoglobin and poor adherence as the major predictors of virologic rebound after treatment simplification.[3] However, these factors were not identified in all trials, suggesting that there are other important determinants of virologic outcomes.[1], [2], [4]–[6]

We assessed the immunologic determinants of sustained virologic suppression in the AIDS Clinical Trials Group (ACTG) protocol A5201. This was a prospective, open-label, single-arm pilot trial of simplified maintenance therapy with atazanavir-ritonavir (ATV/r) alone after prolonged virologic suppression.[7], [8] At week 48, the Kaplan-Meier estimate of the probability of virologic success was 88%.[8] Poor adherence was only documented in 2 patients in the trial and undetectable atazanavir levels were seen in some of the virologic failures in the A5201 study; however, drug levels did not correlate with treatment outcomes. [8] Studies of regimen simplification assume equal recovery of T and natural killer (NK) cells after the CD4^+^ T-cell counts have increased following antiretroviral therapy and levels of HIV-1 RNA have been suppressed for a predetermined time period. The A5201 study used an entry criterion of ≥250 CD4^+^ T cells/mm^3^ to minimize risk of insufficient immune recovery prior to maintenance treatment simplification, similar to other maintenance simplification studies.[5], [8]–[19] We hypothesized that the level of immune activation and recovery of lymphocyte populations influence virologic outcomes for patients undergoing induction-maintenance strategies. Indeed, virologic failure of lopinavir/ritonavir (LPV/r) monotherapy was associated with low nadir CD4^+^ T cell counts and suboptimal medication adherence.[3] In a Swiss study, low nadir CD4^+^ T cell counts were also associated with virologic failure, but in patients that had only been suppressed for 3 months prior to treatment simplification to LPV/r alone.[20] Additionally, trials of treatment simplification have noted increased number of events of HIV-1 viremia above 50 copies/mL as compared to continued combination therapy, which may lead to an increase risk of virologic failure and emergence of antiretroviral resistance.[23], [24]

Our study therefore assessed the immune profile of patients before regimen simplification, the impact of regimen simplification on the T and NK cell populations and immune activation, and whether these immunologic parameters correlated with levels of residual viremia, measured by single copy assay, and detectable viremia above 50 copies/mL.

## Materials and Methods

### Study population

The Institutional Review Boards of all the participating and enrolling institutions listed in the acknowledgements approved the A5201 study and each participant provided written informed consent, these include: the University of Colorado Health Sciences Center, Duke University, Stanford University, the University of Nebraska Medical Center, Weill-Cornell Medical College, the University of Pittsburgh, the University of Cincinnati, the University of Hawaii–Manoa, the University of Iowa, the University of North Carolina–Chapel Hill, the University of Texas–Southwestern Medical Center, and the University of Puerto Rico. Thirty-four participants underwent regimen simplification to ATV/r during the A5201 study. Participants included in the study were receiving a protease inhibitor plus at least 2 NRTIs with plasma HIV-1 RNA suppression below 50 copies/mL for at least 48 weeks immediately prior to study entry.[7] Four participants experienced virologic failure by protocol definition (defined as 2 consecutive plasma HIV-1 RNA levels ≥ 200 copies/mL) at weeks 12, 14, 20 and 28 after simplification. None of these virologic failures developed PI resistance either by standard genotyping or by single genome sequencing.[8] The virologic failures were restarted on triple combination therapy and remained on study until week 54. Of the 34 patients, 25 participants had available cryopreserved PBMC samples for flow cytometry testing, which were collected at entry, and weeks 6, 18, 30 and 54. The 25 participants with available PBMCs did not differ in age, race, nadir or baseline CD4 T cell count, and had been virologically suppressed on average for the same duration as the full study cohort (data not shown). At each time point, 20×10^6^ peripheral blood mononuclear cells (PBMC) were obtained and stored at −140°C. Of the 25 patients with available PBMC samples, one was excluded from the analyses due to poor sample quality (<10% viable PBMC from expected). Most of the PBMC samples were collected at the same timepoints as the HIV-1 viral load samples. All virologic failures as defined in the A5201 clinical trial had available PBMCs and underwent flow cytometry analyses. Clinical trial data including virologic outcomes, CD4 nadir and HIV-1 RNA levels were included in our analyses to assess for potential impact on outcomes or in immune parameters. In all participants, HIV-1 RNA levels were measured with the Roche Amplicor Cobas v1.5 assay. To assess the impact of treatment simplification on HIV-1 residual viremia in patients with long standing HIV-1 suppression, the single copy assay (SCA), having a limit of detection of 1.1 copies/mL as reported by Palmer et al, was used in a subset of 13 participants, which included all 4 participants with study defined virologic failure and 9 participants who had been previously amplified efficiently by SCA in a prior study.[21], [22] In this analysis, we will examine the correlation of persistent HIV-1 residual viremia with immune activation and cell population parameters.

Prior studies of PI monotherapy/simplification using lopinavir/ritonavir have shown an increased prevalence of detectable HIV-1 RNA above 50 copies/mL; persistently detectable HIV-1 RNA levels may potentially lead to virologic failure and emergence of HIV resistance.[23], [24] Therefore, we have used as criteria for detectable HIV-1 RNA any value above 50 copies/mL, which is different from the protocol defined virologic failure of confirmed HIV-1 RNA levels above 200 copies/mL. Eight of the 24 participants tested by flow cytometry had at least one timepoint with HIV-1 RNA >50 copies/mL during the trial, 3 with single, 2 with two consecutive and 3 with multiple detectable timepoints. Seventeen participants had no HIV-1 RNA >50 copies/mL during the trial. We compared immune parameters between the participants with undetectable versus detectable HIV-1 RNA viremia levels during the study.

### Flow cytometric analyses

PBMC samples collected as part of the A5201 protocol at entry, weeks 6, 18, 30 and 54 were used for the analyses. We expanded the flow cytometry testing to include both T cell and NK cell populations, as the latter is associated with early viremic control and may contribute to the control of HIV-1 viremia in patients on ART. The impact of the NK cell population on HIV-1 residual viremia has not been previously evaluated. Cryopreserved PBMC samples were thawed and cell viability was determined using a Vi-CELL analyzer (Beckman Coulter, California) with a target of ≥75% viability for each sample to be used for testing. For each timepoint, 1.5×10^6^ cells were incubated in FACS buffer containing 2%BSA and 0.1% NaN3 along with appropriate combinations of antibodies for 30 minutes following manufacturer's recommendations. The cells were then washed and resuspended in FACS buffer. Flow cytometry data was immediately acquired by using the LSR II flow cytometer (BD Biosciences), in accordance to the manufacturer's instructions. The antibodies used were purchased from BD Biosciences (CD3-PB, anti-CD4-AF700, anti-CD45RO-PE, anti-CD27-FITC, anti-CD69-PC-7, anti-CCR7-APC-AF700, anti-CD38-PERCY5,5, anti-HLADR-APC-H7, anti-CD25-APC, anti-CD95-FITC, anti-PD1-PE, and anti-CD69-PC7, anti-CD16-PERCY5,5, anti-CD158a-FITC, anti-CD158b-PE, anti-CD94-APC, and anti-CD69-PC7), Beckman-Coulter (anti-CD8-ECD), eBioscience (anti-CCR7-APC-AF700) and BioLegend (anti-CD56-AF700). Additional negative controls were included for each sample during staining and acquisition steps. The parent cells were the CD3^+^ T cell lymphocytes for our analyses and all other T and NK cell percentages are based from this parent population. A gate was set on the population of singlet events as determined by the linear relationship between forward scatter height and area. This was followed by a gate on the live population of lymphocytes based on forward and side scatter light properties. The CD56 and CD3 parameters were plotted, and a gate was set on the NK cells based on their classical definition of being CD56+/CD3- lymphocytes. All flow cytometry gating and data were reviewed by both flow cytometry technicians (two) and by the principal investigator to confirm the validity of the samples, the gating strategy and the final numbers, prior to inclusion into the final data set.

### Statistics

All statistical analyses were performed using SPSS statistics 20 (IBM). Sample size was limited to patients participating in the A5201 single arm study with available PBMC samples for analyses. We used both parametric analyses including means test, t-test, and non-parametric analyses including median test, the Mann-Whitney-U, mixed model analyses, and Cox multivariate regression analysis and cumulative survival analyses. Both parametric and non-parametric test were used as appropriate. Univariate analyses were used to examine potential predictors of sustained virologic suppression with p-values ≤ 0.1 which were subsequently analyzed using both forward and backward multivariate regression analysis. Multivariate analyses was used to determine if any of the factors were true predictors of virologic outcome and to eliminate any potential bias or confounding of multiple comparisons/repeated testing producing a false positive finding.[25] We reported the highest significant p-value for the significant variables when examining both the multivariate forward and backward regressions analyses. Statistical significance was defined as P-values <0.05, using a 2-tailed test for multivariate analyses.

## Results

### Baseline immunologic parameters

**Figure 1:**
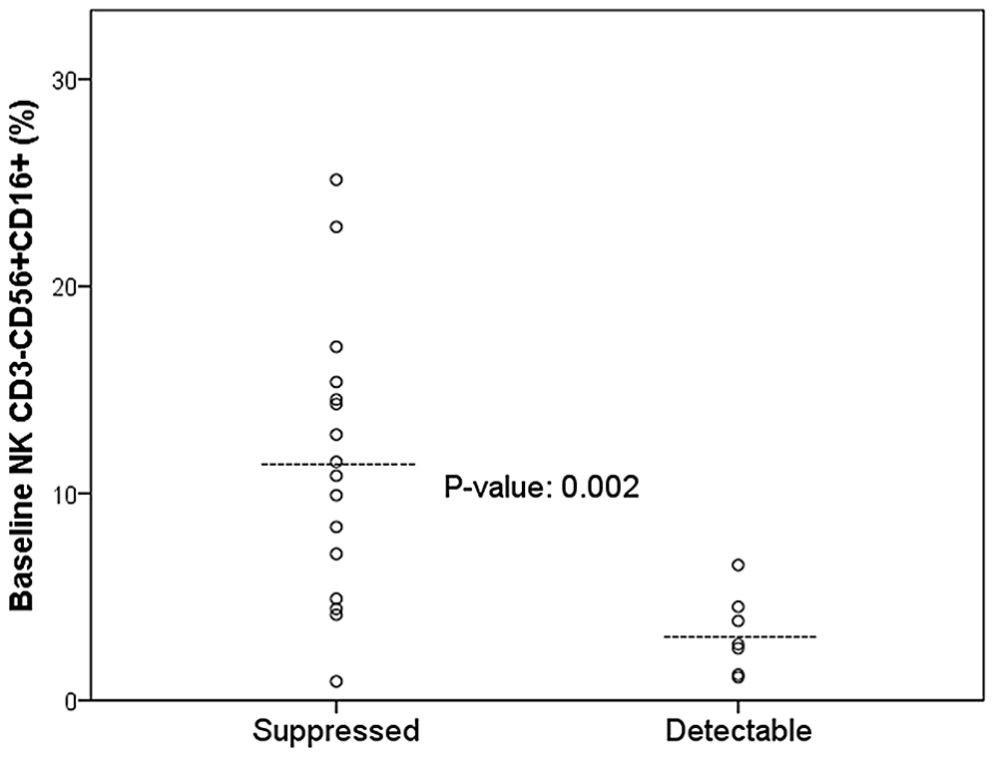
Baseline NK Cell percentages by HIV-1 RNA Outcome following Regimen Simplification. Figure 1 compares the baseline NK cell levels, defined by CD3-CD56+CD16+ cells, and virologic outcome (HIV-1 RNA below or above 50 copies/mL, throughout the study). The circles represent each study participant with either sustained HIV-1 RNA suppression during the trial and those participants who developed detectable viremia following treatment simplification. The difference in the median NK cell levels between the groups with detectable and suppressed viremia was statistically significant with a p-value of 0.002. The median level is noted by the line in the scatterplot for each group.

Participants entering the study had a median CD4^+^ T cell count of 616 (range: 443–756) cells/mm^3^. We compared the baseline T and NK cell parameters between participants maintaining HIV-1 RNA suppression and those who did not (HIV-1 RNA > 50 copies/mL). At baseline, the CD4^+^ and CD8^+^ T cell populations were similar for naïve, central memory, effector memory and CD25^+^ T cells between participants who maintained suppression of HIV-1 RNA and those with detectable HIV-1 RNA (all p-values were non-significant, Table 1). Nadir CD4 cell counts did not correlate with virologic outcomes of detectable HIV-1 RNA above 50 copies/mL or with the assessed immune parameters in multivariable analyses. However, the percentage of CD3^−^ cells that expressed CD56^+^ was significantly higher in participants with undetectable viremia versus those with detectable levels with a mean of 30.6% versus 13.6% (p = 0.03), and for co-expression of CD56^+^CD16^+^ with mean value of 11.5% versus 3.2% (p<0.01). T cells expressing NK cell associated marker CD56^+^ cell levels were also higher in participants with sustained viral load (VL) suppression as compared to participants with detectable VL, with median values of 7.2% versus 4.3% (p<0.01) (Table 1, Figure 1). NK cell baseline differences were statistically significant in both univariate and multivariate analyses including Cox regression (Table 2). Higher NK cell population levels correlated in our analyses with sustained HIV suppression following treatment simplification.

**Table 1:** Univariate analysis of Baseline T and NK Cell Populations and Immune Activation Markers with in Participants with and without detectable HIV-1 RNA after Regimen Simplification.

T & NK cells	HIV-1 Viremia						Median Test	T-test
	UNDETECTABLE (n = 16)			DETECTABLE (n = 8)			Sig. (2-tailed)	Sig. (2-tailed)
	Mean (%)	Std. Deviation	Median (%)	Mean (%)	Std. Deviation	Median (%)		
T cell populations								
CD4^+^	48.07	13.48	48.71	43.81	16.22	50.91	0.52	0.56
CD8^+^	41.13	14.61	42.29	47.25	13.50	44.00	0.36	0.35
CD4^+^CD45RO^−^CCR7^+^	13.26	16.28	8.60	9.61	7.29	6.70	0.58	0.47
CD4^+^CD45RO^+^CCR7^+^CD27^+^	0.23	0.31	0.05	0.91	1.94	0.20	0.17	0.39
CD4^+^CD45RO^+^CCR7^+^CD27^−^	1.70	3.81	0.25	1.49	2.13	0.30	0.89	0.87
CD4^+^CD25^+^	8.75	9.47	4.70	6.16	5.25	4.60	0.51	0.41
CD8^+^CD45RO^−^CCR7^+^	8.08	9.40	5.50	9.73	5.15	10.30	0.67	0.60
CD8^+^CD45RO^+^CCR7^+^CD27^+^	0.04	0.07	0.00	0.04	0.08	0.00	0.90	0.91
CD8^+^CD45RO^+^CCR7^+^CD27^−^	0.31	0.53	0.10	0.24	0.24	0.20	0.76	0.69
CD8^+^CD25^+^	1.41	2.20	0.55	2.00	2.93	0.60	0.60	0.64
NK cell populations								
CD3^−^CD56^+^	30.55	17.52	27.37	13.62	9.53	9.58	**0.03**	**0.01**
CD3^−^CD56^+^CD16^+^	11.52	6.70	11.19	3.21	1.92	2.71	**0.00**	**0.00**
CD3^+^CD56^+^	11.52	11.71	7.18	3.76	1.92	4.31	**0.10**	**0.02**
IA Markers: CD4+ T cells								
CD4^+^CD38^+^	31.59	14.26	33.50	29.01	12.48	28.60	0.68	0.67
CD4^+^HLADR^+^	23.93	26.52	13.55	4.99	2.07	4.20	0.08	**0.01**
CD4^+^CD69^+^	21.25	22.47	12.15	6.67	1.54	6.30	0.11	**0.02**
CD4^+^CD95^+^	45.58	13.76	45.95	39.21	15.43	42.50	0.34	0.37
CD4^+^CD279^+^	1.80	5.79	0.30	1.47	2.59	0.70	0.89	0.85
CD4^+^CD38^+^HLADR^+^	5.61	7.95	3.55	1.57	1.27	1.20	0.20	0.07
IA Markers: CD8+ T cells								
CD8^+^CD38^+^	10.80	7.82	8.05	9.69	5.31	8.60	0.74	0.70
CD8^+^HLADR^+^	20.29	16.05	16.80	6.74	2.66	6.10	**0.04**	**0.00**
CD8^+^CD69^+^	15.49	19.13	8.35	3.99	2.44	3.70	0.13	**0.03**
CD8^+^CD95^+^	37.88	17.26	35.95	48.83	15.34	52.10	0.16	0.15
CD8^+^CD279^+^	0.21	0.23	0.10	0.77	0.89	0.40	**0.02**	0.15
CD8^+^CD38^+^HLADR^+^	2.96	3.21	1.90	1.81	0.91	1.60	0.37	0.21
IA Markers: NK cells								
CD3^−^CD56^+^CD69^+^	31.44	20.38	27.04	17.06	10.36	15.86	0.09	**0.04**
CD3^−^CD56^+^CD94^+^	55.85	18.92	55.49	54.19	23.43	62.73	0.86	0.87
CD3^−^CD56^+^CD158a^+^	30.11	19.75	33.11	21.78	15.41	22.42	0.33	0.29
CD3^−^CD56^+^CD158b^+^	25.71	19.13	30.51	37.05	23.45	45.51	0.23	0.29

The significant p-values for univariate comparisons are in bold.

**Table 2:** Univariate and Multivariate Regression Analysis of Baseline T and NK Cell Populations and Immune Activation Markers and the Risk of Detectable Plasma HIV-1 RNA.

Cox Regression Analysis				

**Cell Markers**	**Univariate (p-value)**	**Univariate OR (95% CI)**	**Multivariate (p-value)**	**Final OR (95% CI)**
CD4^+^ CD69^+^	0.071	13.2 (1.24–140.69)	NS	
CD4^+^ DR^+^	0.093	4.2 (0.60–28.62)	NS	
CD4^+^ CD38^+^ DR^+^	0.039	13.2 (1.24–140.69)	NS	
CD8^+^ CD69^+^	0.033	4.2 (0.60–28.62)	NS	
CD8^+^ DR^+^	0.045	13.2 (1.24–140.69)	NS	
CD8^+^ CD279^+^	0.1	0.13 (0.01–1.34)	NS	
CD3^+^ CD56^+^	0.012	13.2 (1.24–140.69)	NS	
CD3^−^ CD56^+^	0.033	13.2 (1.24–140.69)	NS	
CD3^−^ CD56^+^ CD16^+^	0.007	13.2 (1.24–140.69)	**0.043**	10.3 (1.92–55.3)
CD3^−^ CD56^+^ CD69^+^	0.053	4.2 (0.60–28.62)	0.055	
CD3^−^ CD56^+^ CD158a^+^	0.093	1.71 (0.29–10.30)	NS	

Cox regression analyses included all variables with p-values ≤0.1 in the univariate analyses, and age, race and CD4 nadir prior to starting ART. Odds ratio above 1.0 are associated with increased risk of virologic failure.

Significant differences in T cell immune activation at baseline were seen between the two groups. CD4^+^CD38^+^HLADR^+^, CD8^+^CD69^+^, and CD3^−^CD56^+^CD69^+^ percentages were higher in the participants whose HIV-1 RNA levels remained suppressed below 50 copies/mL during the study. In comparison of participants with undetectable versus detectable viremia, the median percentages for CD4^+^HLADR were 13.6% vs. 4.2% (p = 0.01), CD4^+^CD69+ were 12.2% versus 6.3% (p = 0.02), CD4^+^CD38^+^HLADR^+^ were 3.6% versus 1.20% (p = 0.07), CD8^+^HLADR^+^ were 16.8% versus 6.1% (p<0.01), CD8^+^CD69^+^ were 8.4% versus 3.7% (p = 0.03) and for CD56^+^CD69^+^ were 27.0% versus 15.9% (p = 0.04) (Table 1). The lower T cell immune markers may represent early migration of activated T cells from peripheral circulation to the tissues where active HIV replication is ongoing in participants with detectable viremia.

### Association of baseline immunologic status with virological outcome

**Figure 2:**
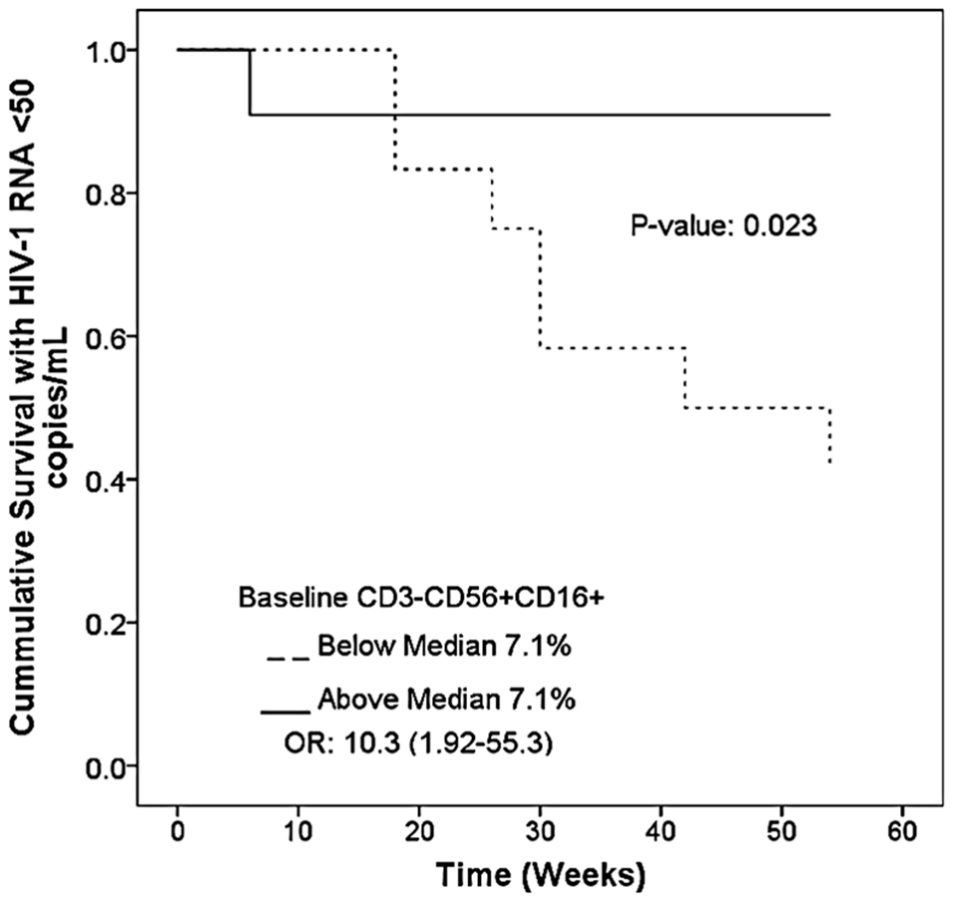
Proportions with HIV-1 RNA < 50 copies/ml after regimen simplification by baseline NK cell levels. Figure 2 shows a Kaplan-Meier plot for the survival with HIV-1 RNA level above 50 copies/mL during the A5201 study based on the participant's NK cell level at study entry. The p-value is 0.023 by Kaplan-Meier analyses for the difference at 54 weeks between the groups above and below the median NK cell level.

Cox univariate regression analyses of baseline immunologic parameters as continuous variables were performed to assess for any associations with the development of detectable HIV-1 RNA levels above 50 copies/mL. Only 9 of the T or NK cell populations or immune activation parameters analyzed had a p-value of ≤0.1 in univariate regression, including CD4^+^CD69^+^, CD4^+^CD38^+^HLA-DR^+^, CD8^+^HLA-DR^+^, CD8^+^CD69^+^, CD8^+^CD279^+^, CD3^+^CD56^+^, CD3^−^CD56^+^, CD3^−^CD56^+^CD16^+^, CD3^−^CD56^+^CD69^+^ and CD3^−^CD56^+^CD158a^+^. These immunologic variables were included in a multivariable regression analysis with forward and backward regression. We also included participant demographic characteristics, including age and race, and nadir CD4^+^ T cell count prior to start of antiretroviral treatment. Only one parameter-population remained in the model, CD3^−^CD56^+^CD16^+^, with a p-value of 0.043 in forward regression and 0.034 in backwards regression analyses. NK cells expressing the CD69^+^ cell-associated marker of early activation did not remain in the model but the p-value neared statistical significance at 0.055 (Table 2). Cumulative survival analyses of the impact of NK cells levels, as a dichotomous variable, determined that participants with NK cell levels above the median of 7.1% had reduced risk of developing detectable HIV-1 viremia above 50 copies/mL as compared to those with NK cell levels below the median (p =  0.023) (Figure 2). The odds ratio of detectable HIV-1 RNA viremia in participants with low NK cell levels was 10.3 (95% CI: 1.92 to 55.3). Independent of baseline levels of immune activation and other T cell populations, the baseline NK cell level was the only predictive marker of detectable HIV-1 viremia in multivariate regression following treatment simplification. Interestingly, CD8^+^CD38^+^HLA-DR^+^ levels were statistically significantly different at week 30 of the study in participants with and without detectable viremia (p =  0.034) but the difference was not present by week 54 (p = 0.67).

### Correlation of T and NK cell populations and Immune Activation (IA) Markers with HIV-1 RNA levels

Levels of T and NK cell populations and immune activation markers were analyzed with HIV-1 RNA levels using both the Single Copy Assay (SCA) and/or the Roche Amplicor v1.5 assay. During the trial, blood samples with detectable HIV-1 RNA levels below 50 copies/mL by SCA correlated with higher NK cells expressing CD158b^+^ and CD94^+^ markers (r =  0.455 & 0.375, p-values =  0.006 & 0.027, respectively). Other parameters were not significantly correlated (data not shown).

**Figure 3:**
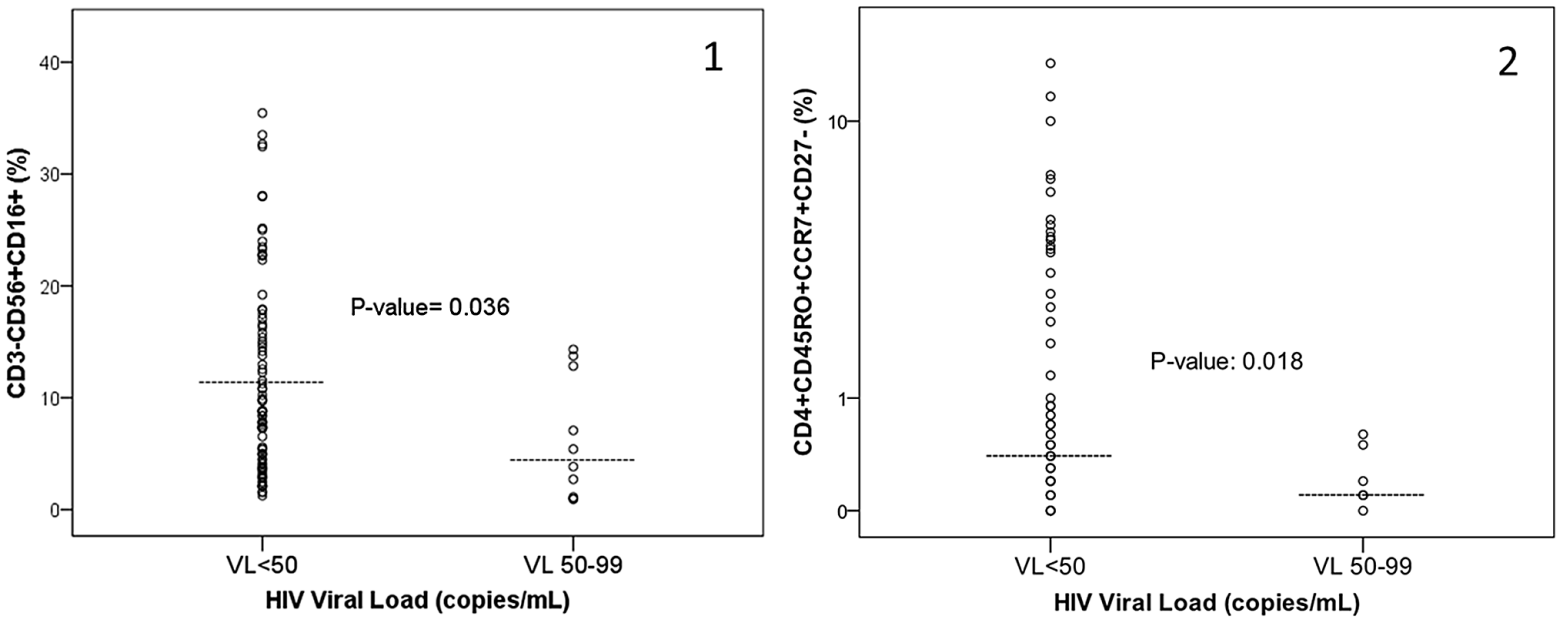
Comparison of NK cells and T cell populations based on HIV-1 viremia below and above 50 copies/mL. Figure 3 demonstrates that the population percentage of CD3-CD56+CD16+ cells (panel 1) and CD4-CD45RO+CCR7+CD27- cells (panel 2) were decreased in samples with HIV-1 RNA levels of 50–99 copies/mL as compared to samples obtained with HIV-1 RNA below 50 copies/mL with p-values of 0.036 and 0.018, respectively. The median level for each cell population is indicated by the line in the scatterplot for each group.

When comparing samples with HIV-1 RNA levels 50–99 copies/mL to those below 50 copies/mL by Roche Amplicor v1.5, we determined that the higher viral load group had lower median levels of CD4^+^CD45RO^+^CCR7^+^CD27^−^ (0.1% vs. 0.4%, p = 0.018), CD8^+^CD38^+^HLA-DR^+^ (1.3% vs. 1.8%, p = 0.013), NK cells CD3^−^CD56^+^CD16^+^ (4.6% vs. 11.5%, p = 0.036) and CD56^+^CD94+ (38.3% vs. 59%, p = 0.005), in univariate analyses (Figure 3). Other T and NK cell populations and IA markers were not significantly different when compared at different HIV viral load levels. In multivariate analyses, only NK cell levels remained significant with detectable viremia (Table 2).

### Impact of treatment simplification on T and NK cell populations and IA

Comparisons of the T and NK cell populations and levels of IA markers before and after treatment simplification revealed no impact of treatment simplification on the immune parameters examined. Participants with suppressed viremia during the study tended to have an increase in the percentage of CD4^+^CD25^+^ cells with treatment simplification (p = 0.08), but by study week 54 the levels were similar to baseline (p = ns). The CD4^+^CD45RO^+^CCR7^+^CD27^−^ cell population also showed a trend to increase following simplification in the participants with suppressed viremia during the study but did not reach statistical significance (p = 0.073). No statistically significant difference was seen in immune activation levels following treatment simplification in either outcome group. All other T and NK cell markers were not statistically associated with treatment simplification (all p-values >0.08 (data not shown).

## Discussion

We examined the impact of baseline T and NK cell populations and markers of immune activation on virologic outcomes following regimen simplification to atazanavir/ritonavir alone. In addition, the impact of regimen simplification on immunologic parameters was investigated. Our study is the first to measure a possible predictive value of baseline NK cell levels for virologic outcome. Participants with NK cell levels below the median had 10 times higher risk of developing HIV-1 RNA levels above 50 copies/mL during the study. The difference in NK cell levels was seen in our study group while patients were still on their original combination antiretroviral therapy and before any intervention had occurred. Increased incidence of detectable viremia above 50 copies/mL has been reported in other trials of treatment simplification strategies and has led to a concern for increased risk of virologic failure and possible development of drug resistance mutations. [16], [23], [24], [26] Therefore, understanding the immunologic control of low level viremia in participants undergoing treatment simplification and selecting a stricter 50 copies/mL threshold was considered to be more informative than using the study defined confirmed virologic failure above 200 copies/mL for our analyses.

The NK cell levels and immune activation marker differences at baseline demonstrate that despite prolonged HIV-1 RNA suppression on antiretroviral therapy, participants had significant differences in immunologic parameters at entry into the study. Therefore the immune status of patients entering HIV clinical trials as currently assessed by CD4^+^ T cell levels prior to study entry does not provide a complete assessment of functional immune status reconstitution, and duration of HIV-1 RNA suppression may not be sufficient as an entry criterion for trials of maintenance therapy. Other parameters that were associated with detectable HIV-1 viremia at some point during the study included markers of immune activation HLA-DR and CD38, which could be indicators of undetected viral replication and/or ongoing inflammation, and in turn impact treatment outcomes. These markers reached statistical significance in univariate analyses but did not remain in the model as significant factors in multivariate analyses, possibly due to the limited sample size of our study. Larger studies are needed to further evaluate if the cell markers not retained in the regression models were affected by the limited sample size and have a significant impact on ART treatment responses.

Regimen simplification to atazanavir/ritonavir was not found to be an independent predictor of changes in either T or NK cell populations or on the levels of immune activation markers. We observed initial gains in CD4^+^ naïve and CD4^+^CD25^+^ T cells in participants with virologic suppression during the trial but the differences were not sustained after 48 weeks of simplification. In the OK04 trial regimen simplification to LPV/r was associated with a modest increase in CD4^+^ T cells at 48 weeks.[6] CD4^+^ T cells increases were not detected in our study as previously reported.[8]

HIV-1 residual viremia below 50 copies/mL and low level viremia between 50–100 copies/mL were associated with distinct changes in immune cell populations and activation markers. NK cell CD158b^+^ and CD94^+^ surface cell markers tended to increase with rising residual viremia by SCA. Once the HIV-1 RNA levels were between 50–99 copies/mL, then CD4^+^ central memory T cells, NK cells and markers of inflammation tended to decrease as compared to samples from participants with suppressed HIV-1 RNA levels. These paradoxical decreases in markers of inflammation could be related to migration of activated cells from the circulation to the periphery as HIV-1 viral replication expands. These findings may be relevant as we monitor patients with low level detectable viremia or viral blips, as these could signify that the low level increase in plasma HIV-1 RNA is due to actual replication instead of assay variability.

Both the innate and adaptive immune systems have a role in the host response against HIV-1 viral replication. Initial viremia control in acute infection is achieved by cytotoxic activity of CD8^+^ T and NK cell responses.[27], [28] NK cells are known to be involved in both the early antiviral response to HIV-1 infection and represent an important part of innate and possibly adaptive immune response to control HIV-1 viral replication.[27]–[32] Concomitant CD4^+^ and CD8^+^ T cell responses to Gag peptides and NK cell responses to Env and Reg peptides have been correlated with improved viremia control and higher CD4^+^ T cell counts.[33] NK cell responses to HIV-1 peptides have been demonstrated to persist even in absence of CD4^+^ and CD8^+^ T cell responses.[33] NK cell population levels and the phenotypic changes identified in our study suggest a more robust role of these cells in functional and cytotoxic activities related to control of HIV-1 replication in patients with detectable viremia below 50 copies/mL. Prior studies have shown that either changes in NK cell phenotype or functionality can impact HIV-1 viral control when assessed in long-term non-progressors and HIV-1 controllers.[34], [35] Decreases in CD56^bright^ subsets, NKp30 and NKp46 expression in NK cells have been associated with reduced activation and cytotoxic activity with progressive HIV-1 infection.[36]–[39] NK cell exhaustion may also be associated with decreased viremic control, as HIV controllers expressed normal Siglec-7 levels as compared to HIV-1 progressors.[40] Similarly, PBMCs obtained from of long term non-progressors show normal to low expression of different inhibitory natural killer receptors (iNKR) in CD3+CD8+ CTL cells and lack of inhibition of HIV-1 specific cytotoxic activity by iNKR in vitro suggesting that normalized receptor expression may be needed for on-going anti-HIV cytotoxic activity.[41] Virologic failure may be as dependent on the host innate immune responses beyond just CD4^+^ T cell recovery as it is on regimen potency.

The A5201 study was designed as a single arm study, which limits our ability to compare our results to control participants not undergoing treatment simplification. SCA HIV-1 viral load data are also limited to 13 participants who were known to have amplifiable HIV-1 gag sequences, limiting the depth of the analyses below 50 copies/mL. Low level detectable HIV-1 viremia above 50 copies/mL seen in this study may represent either new full rounds of replications due to incomplete suppression or viral shedding from previously infected cells. In the A5201 study, no PI resistance mutations were detected by either standard or single genome sequencing to support possible viral evolution and therefore new rounds of replication. Poor medication adherence will impact virologic rebound and was documented in at least two A5201 study participants. However, most study participants indicated good medication adherence during the trial and had been suppressed for longer to 12 months prior to study entry.[8] Atazanavir drug levels were evaluated in the original study but were not correlated with virologic outcomes, except if no level was detected (data not shown). Study medication adherence is unlikely to have impacted baseline T and NK populations prior to study intervention, unless inconsistent medication adherence had been unrecognized prior to study entry and had not resulted in virologic rebound. Due to the original study sample size and sample availability, our final sample size is relatively small and some of the variables tested may not have reached statistical significance due to a type B error. Larger studies will be needed to further assess our study results and elucidate the impact of other immunologic parameters on HIV-1 viremic control. The strength of our study, however, lies in the depth and robustness of the immunologic flow cytometric analyses which included T and NK cells, together with correlation with clinical outcomes and HIV-1 RNA levels from both single copy assay and standard viral load testing.

In summary, this study demonstrates that patients on suppressive ART for similar timeframes have distinct immune cell populations and levels of cellular activation, despite CD4^+^ T cell counts on average above 600 cells/mm^3^, and even after adjusting for nadir CD4^+^ T cell counts. Different levels of immune reconstitution and activation may explain the differences in treatment outcomes in trials of maintenance regimen simplification, and could explain why some patients may not require triple combination therapy to maintain HIV-1 suppression. Future trials of maintenance therapy strategies should include more in-depth immune assessments to further confirm these findings and better understand the relationship between the NK cell populations and other immune markers and treatment outcomes. Baseline assessment of NK cell levels and immune activation markers could provide information for early detection of patients at increased risk of virologic failure.
